# The “Healthcare Workers’ Wellbeing (Benessere Operatori)” Project: A Picture of the Mental Health Conditions of Italian Healthcare Workers during the First Wave of the COVID-19 Pandemic

**DOI:** 10.3390/ijerph18105267

**Published:** 2021-05-15

**Authors:** Valentina Elisabetta Di Mattei, Gaia Perego, Francesca Milano, Martina Mazzetti, Paola Taranto, Rossella Di Pierro, Chiara De Panfilis, Fabio Madeddu, Emanuele Preti

**Affiliations:** 1 School of Psychology, Vita-Salute San Raffaele University, 20132 Milan, Italy; dimattei.valentina@hsr.it; 2 Clinical and Health Psychology Unit, IRCCS San Raffaele Scientific Institute, 20132 Milan, Italy; f.milano1@studenti.unisr.it (F.M.); mazzetti.martina@hsr.it (M.M.); taranto.paola@hsr.it (P.T.); 3 Department of Psychology, University of Milan-Bicocca, 20126 Milan, Italy; rossella.dipierro@unimib.it (R.D.P.); fabio.madeddu@unimib.it (F.M.); emanuele.preti@unimib.it (E.P.); 4 Department of Medicine and Surgery, University of Parma, 43126 Parma, Italy; chiara.depanfilis@unipr.it

**Keywords:** COVID-19, mental health, health care, health professions, depression, anxiety, insomnia, post-traumatic stress, anger, burnout

## Abstract

During the last year, the COVID-19 outbreak put all the healthcare workers around the world at risk of physical and psychological sequelae. The general purpose of the present study was to assess the mental health of Italian healthcare workers during the COVID-19 outbreak and to identify high-risk groups. Here, we present results from the baseline assessment of the “Healthcare workers’ wellbeing (Benessere Operatori)” project on a sample of 1055 healthcare workers. Participants completed the Depression Anxiety Stress Scale-21, the Insomnia Severity Index, the Impact of Event Scale-Revised, the State-Trait Anger Expression Inventory-2, and the Maslach Burnout Inventory. Healthcare workers who worked in COVID wards reported higher levels of anxiety, insomnia, post-traumatic stress, anger, and burnout, compared to those reported by the healthcare workers who worked in non-COVID wards. Moreover, nurses, both in COVID and non-COVID wards, were at higher risk of experiencing psychological distress compared to other groups of healthcare workers. These findings highlight the importance of implementing targeted psychological interventions for healthcare workers operating in COVID wards and nurses, who seem to be the most vulnerable categories.

## 1. Introduction

The World Health Organization (WHO) declared the coronavirus disease 19 (COVID-19) outbreak a global pandemic on 11 March 2020. The pandemic rapidly spread to over 200 countries in the subsequent months, and millions of healthcare workers around the world have been employed on the front line to save lives and reduce the risk of virus transmission. All of them have been working in a challenging situation and experiencing great psychological distress [1,2,3].

Previous research already documented the difficult conditions healthcare workers face during epidemics, highlighting the presence of work-related stress, post-traumatic stress, depression, insomnia, anxiety, and general psychiatric symptoms [4], with increasing evidence suggesting that COVID-19 may be the most common independent risk factor for stress, psychological distress, and post-traumatic stress disorder (PTSD) in healthcare workers [5,6,7,8].

Notably, COVID-19 puts healthcare professionals in an unprecedented condition, forcing them to make difficult decisions and work under extreme pressure. These decisions entail distributing scarce resources to similarly vulnerable patients, reconciling their own physical and mental health needs with those of patients, matching their desire and responsibility to patients with those of family and friends, and treating all seriously ill patients with limited or inadequate resources [2]. Moreover, the non-quantifiable numbers of patients with critical conditions, the unpredictable course of the disease, the high morbidity and mortality rates, and the lack of defined therapies have generated feelings of fear and helplessness in healthcare workers, severely affecting their mental health conditions [5,7] and causing feelings of irritability, frustration, and anger [9]. In particular, increased workload and job deterioration could exacerbate healthcare workers’ state of anger [10,11]

Therefore, healthcare workers are at risk not only of detrimental physical effects from COVID-19, but also of harmful psychological sequelae [12,13]. On the one hand, the literature reports several risk factors for healthcare workers’ mental health, including female gender, age <40, low income, a higher number of family/home stressors, isolation from family, poor supervisor and organizational support, high workload, working in unsafe settings, time pressure, working with COVID-19 patients, being part of the nursing staff, fewer years of working experience, history of organic and mental illness, higher avoidance strategies, and lower positive attitude [5,6,7,14,15,16]. On the other hand, the presence of children, a strong social and family network, team cohesion and shared responsibility among colleagues, adequate personal protective equipment, the use of humor and planning as coping strategies, and the ability to talk to someone about their experiences seem to be protective factors for healthcare workers’ mental health [5,6,14,15,16]. Similar findings have been reported in Italian samples [17,18,19,20,21,22].

Furthermore, the literature extensively highlights differences in mental health among different categories of healthcare workers, both during previous epidemics [4,5] and during the COVID-19 outbreak [7,15,23,24]. In particular, nurses reported higher levels of anxiety, depression, and PTSD symptoms than did other groups of healthcare workers and clerical staff [5,7,15,24]. These differences are probably related to the amount of time spent in contact with patients [25].

In light of the extreme importance of this topic, we conducted a web-based longitudinal survey to examine the psychological impact of the COVID-19 pandemic on a sample of Italian healthcare workers involved in the management of the pandemic. In this contribution, we present baseline findings of the “Healthcare workers’ wellbeing (Benessere Operatori)” project. The project consists of a first baseline assessment, conducted between 9 May 2020 and 13 July 2020, aimed at collecting data on levels of psychological distress, as well as on related socio-demographic, situational, and personal factors that can affect individuals’ psychological response to the COVID-19 pandemic to identify high-risk groups. A second phase of the study was conducted in December 2020 and a third phase will be conducted at the end of May 2021 to test long-term psychological consequences of the pandemic.

## 2. Materials and Methods

### 2.1. Participants and Procedure

The study was conducted according to the guidelines of the Declaration of Helsinki and approved by the Ethics Committee of the University of Milan-Bicocca (protocol n. 0024531/20), the Ethics Committee of the IRCCS San Raffaele Scientific Institute (protocol n. 109/2020), and the Ethics Committee of the Parma Local Health Authority (protocol n. PG0019826_2020).

We conducted a baseline assessment between 9 May and 13 July 2020, after the main peak of COVID-19 outbreak in Italy. We spread information about the study (www.benessereoperatori.com, accessed on 9 May 2020) by contacting national healthcare workers’ professional boards and associations, posting pamphlets inside hospitals, and through campaigns on social networks. After reading the informed consent, participants voluntarily completed an online survey administered through Qualtrics. We assessed participants’ socio-demographic characteristics, working conditions, individual perception of the COVID-19 situation, anxiety, depression, and insomnia symptoms, post-traumatic stress, state anger, and burnout levels. The baseline battery also included a measure of coping strategies and perceived social support. However, we did not examine these variables in the current work.

The initial dataset included 1090 participants. Thirty-five participants declared that they had not been working during the past three months; therefore, they were excluded from the analyses, resulting in a final sample of 1055 participants (75.70% females, *n* = 799) with an overall mean age of 44.74 years (*SD* = 11.30, range = 20–79). Forty-nine percent (*n* = 517) of them were married and 57.10% (*n*=602) had children. Only 16.70% (*n* = 176) were living alone. Most of the participants (*n* = 1033, 98.10%) were located in one of the four regions most affected by the COVID-19 outbreak (i.e., Lombardy, Emilia-Romagna, Piedmont, and Veneto), while the remaining participants (*n* = 22, 1.90%) were located in other regions. About 22.40% (*n* = 236) of the sample reported a mental health disorder in the past. Although 18% (*n* = 189) of the sample reported being at least somewhat likely to need psychological or psychiatric support, only 5.10% (*n* = 54) reported being at least somewhat likely to seek psychological or psychiatric support during the next week.

Concerning their occupation, 28.20% (*n* = 298) of our participants were physicians, 34.30% (*n* = 362) were nurses, 24.70% (*n* = 261) were other healthcare workers (i.e., psychologists, physiotherapists, healthcare assistants, midwives, radiology technicians, laboratory technicians, psychiatric rehabilitation technicians, speech therapists, social workers, and biologists), and 12.70% (*n* = 134) were clerks. About 45.60% (*n* = 481) of them had more than 20 years of professional experience and 23.60% (*n* = 249) reported having felt very likely or extremely likely at risk of making mistakes during the previous three months. Among healthcare workers, 35.20% (*n* = 332) worked in COVID-19 wards.

Finally, 25.90% (*n* = 273) of our participants reported having symptoms of COVID-19, and 21% (*n* = 222) and 81.40% (*n* = 859) reported having relatives and colleagues with symptoms of COVID-19, respectively.

### 2.2. Measures

The DASS-21 [26,27] is a 21-item measure, evaluating general distress on a tripartite model of psychopathology. This questionnaire is divided into three subscales: depression, anxiety, and stress. Each subscale contains seven items rated on a 4-level Likert scale (0 = never; 3 = almost always). The total score is estimated by adding together the response values of each item. Higher scores indicate severe levels of depression, anxiety, and stress symptoms (α range = 0.85–0.95). The score at the depression subscale (e.g., “I felt I wasn’t worth much as a person”) is grouped into normal (0–9), mild (10–12), moderate (13–20), severe (21–27), and extremely severe depression (28–42). The score at the anxiety subscale (e.g., “I felt scared without any good reason”) is split up into normal (0–6), mild (7–9), moderate (10–14), severe (15–19), and extremely severe anxiety (20–42). The score at the stress subscale (e.g., “I found it difficult to relax”) is divided into normal (0–10), mild (11–18), moderate (19–26), severe (27–34), and extremely severe stress (35–42) [28].

The Insomnia Severity Index (ISI) [29,30] is a self-report questionnaire evaluating the nature, severity, and impact of insomnia through seven items rated on a 5-level Likert scale (0 = “no problem”; 4 = “very severe problem”). Scores range from 0 to 28 and can be classified into absence of insomnia (0–7); sub-threshold insomnia (8–14); moderate insomnia (15–21); and severe insomnia (22–28). The dimensions assessed include severity of sleep onset, sleep maintenance, early morning awakening problems, sleep dissatisfaction, interference of sleep difficulties with daytime functioning, noticeability of sleep problems by others, and distress caused by the sleep difficulties (α = 0.62).

The Impact of Event Scale-Revised (IES-R) [31,32] is a 22-item self-report questionnaire assessing the frequency of intrusive and avoidant thoughts and behaviors associated with a traumatic event. Items are rated on a 5-points Likert scale (0 = “not at all”; 4 = “extremely”). The IES-R is composed of three subscales. Intrusion (8 items) measures intrusive thoughts, nightmares, intrusive feelings, and imagery associated with the traumatic event; avoidance (8 items) measures avoidance of feelings, situations, and ideas; hyperarousal (6 items) measures difficulty in concentrating, anger and irritability, psychophysiological arousal upon exposure to reminders, and hypervigilance (α range = 0.85–0.95).

The State-Trait Anger Expression Inventory-2 (STAXI-2) [33,34] is a 57-item self-report questionnaire measuring five domains of anger: State-Anger, Trait-Anger, Anger Expression-In, Anger Expression-Out, and Anger-Control. Responses are rated on a 4-point Likert scale, ranging from 1 (not at all) to 4 (almost always). In order to measure healthcare workers’ acute reaction to the pandemic, we only administered the State-Anger subscale, which is composed of three subscales: Feeling Angry (5 items), Verbal Expression (5 items), and Physical Expression (5 items) (α range = 0.89–0.94).

The Maslach Burnout Inventory (MBI) [35,36] is composed of 22 items divided into three subscales assessing the three components of the burnout syndrome: emotional exhaustion (9 items), depersonalization (5 items), and personal accomplishment (8 items). Each item is rated on a 7-point Likert scale (0 = “never”; 6 = “every day”) (α range = 0.75–0.93). For Italian healthcare workers, subscales scores can be classified as follows: the emotional exhaustion subscale scores are divided into low (≤14), medium (15–23), and high (≥24); the depersonalization subscale scores are grouped into low (≤3), medium (4-8), and high (≥9); and the personal accomplishment subscale scores are classified into low (≥37), medium (30-36), and high (≤29) [36].

Moreover, we measured how much participants were worried about the possibility that they, their relatives, and their colleagues could become infected by COVID-19. Four items were rated on a 5-point Likert scale (1 = “not at all”; 5 = “extremely”). A total score of worry was obtained by averaging items scores. Higher scores indicate higher levels of worry (α = 0.86).

Finally, we measured participants’ working conditions during the past three months in several areas, including eating, sleeping, working shifts, being isolated, and wearing adequate protective equipment. Seven items were rated on a 5-point Likert scale (1 = “not at all”; 5 = “very much”). A total score of working conditions was obtained by averaging items scores. Higher scores indicate worse working conditions (α = 0.69).

### 2.3. Statistical Analysis

Psychological scales were summarized using median and interquartile range (IQR), while categorical variables were reported by means of frequency distribution and percentages. Since psychological scales showed skewed distributions, the Kruskal–Wallis test was applied for comparing scores obtained by different working categories. In the presence of a significant Kruskal–Wallis result (*p* < 0.05), a post-hoc analysis was performed to examine specific categories of interest taken in pairs (Dunn’s test was performed followed by Bonferroni’s correction for adjusting for multiple testing). The following comparisons were considered relevant for the study: COVID physicians vs. non-COVID physicians; COVID nurses vs. non-COVID nurses; COVID other vs. non-COVID other; COVID physicians vs. COVID nurses; COVID physicians vs. COVID other; non-COVID physicians vs. non-COVID nurses; non-COVID physicians vs. non-COVID other; and clerks were compared with all the aforementioned groups.

## 3. Results

As listed in Table 1, the Kruskal–Wallis test showed a significant difference in the distribution of scores for the different groups in the following variables: Anxiety (*p* < 0.0001) and Stress (*p* = 0.0275) subscales of the DASS-21; ISI total score (*p* < 0.0001); Intrusion (*p* < 0.0001), Avoidance (*p* < 0.0001), and Hyperarousal (*p* = 0.0003) subscales of the IES-R; State-Anger (*p* = 0.0012), Feel like expressing Anger Verbally (*p* < 0.0001), and Feel like expressing Anger Physically (*p* < 0.0001) subscales of the STAXI-2; Emotional Exhaustion (*p* < 0.0001), Depersonalization (*p* < 0.0001), and Personal Accomplishment (*p* < 0.0001) subscales of the MBI; Working conditions (*p* < 0.0001); and Worry (*p* = 0.0403).

### 3.1. Depression, Anxiety, and Stress Symptoms

With reference to the cut-off values reported in the literature [26] (see Table 2), more than 65% of the sample reported normal or mild scores of depression, anxiety, and stress symptoms. However, 12.21% of non-COVID nurses, 12.63% of COVID nurses, and 16.28% of other COVID healthcare workers reported extremely severe anxiety symptoms. Only 1.5% of non-COVID physicians, 4.08% of COVID physicians, 4.13% of other non-COVID healthcare workers, and 4.48% of clerks reported extremely severe anxiety symptoms.

Considering the whole set of scores (see Supplementary Material, Figure S1 for a graphical representation), there was a significant difference in the distribution of anxiety scores between non-COVID nurses and non-COVID physicians (Dunn’s test *p* = 0.0019); between non-COVID nurses and other non-COVID healthcare workers (Dunn’s test *p* = 0.0017); between COVID nurses and COVID physicians (Dunn’s test *p* = 0.0003); and between COVID nurses and clerks (Dunn’s test *p* = 0.0328).

### 3.2. Insomnia Symptoms

With reference to the cut-off values reported in the literature [30], more than 85% of the sample reported absent or sub-threshold insomnia; however, 12.21% of non-COVID nurses and 13.16% of COVID nurses showed moderate insomnia. Only one COVID nurse reported a severe level of insomnia. A total of 1.5% of non-COVID physicians reported moderate insomnia while 5.1% of COVID physicians reported moderate insomnia. None in the “physician” category reported severe levels of insomnia. Considering the whole set of scores, there was a significant difference in the distribution of insomnia scores (see Figure S2) between non-COVID nurses and non-COVID physicians (Dunn’s test *p* = 0.0051); between COVID nurses and COVID physicians (Dunn’s test *p* = 0.0148); and between COVID nurses and clerks (Dunn’s test *p* = 0.0129).

### 3.3. Post-Traumatic Stress Symptoms

Considering the whole set of scores, there was a significant difference in the distribution of intrusion, avoidance, and hyperarousal (see Figures S3–S5 respectively) scores between non-COVID nurses and non-COVID physicians (*p* = 0.0009; *p* = 0.0001; *p* = 0.0216, respectively); and between non-COVID nurses and other non-COVID healthcare workers (Dunn’s test *p* = 0.0011; *p* = 0.0083; *p* = 0.0363, respectively). Moreover, there was a significant difference in the distribution of intrusion scores between COVID physicians and non-COVID physicians (Dunn’s test *p* = 0.0047); and between COVID nurses and clerks (Dunn’s test *p* = 0.0004). From a purely descriptive perspective, the highest percentage of extremely severe scores was recorded for COVID nurses (5.79%), followed by COVID physicians (5.1%).

### 3.4. State-Anger

Considering the whole set of scores, there was a significant difference in the distribution of State-Anger scores (see Figure S6) between COVID physicians and non-COVID physicians (Dunn’s test *p* = 0.0471). Moreover, there was a significant difference in the distribution of verbal expression of anger (see Figure S7) between COVID nurses and clerks (Dunn’s test *p* = 0.0473). There was also a significant difference in the distribution of physical expression of anger (see Figure S8) between COVID nurses and non-COVID nurses (Dunn’s test *p* = 0.0167).

### 3.5. Burnout Symptoms

With reference to the cut-off values reported in the literature [36], more than 28% of the sample reported high levels of emotional exhaustion, except for other non-COVID healthcare workers (19.27%). Actually, post-hoc analysis highlighted a significant difference in the distribution of emotional exhaustion scores (see Figure S9) when comparing other non-COVID healthcare workers to both non-COVID physicians (Dunn’s test *p* = 0.0003) and non-COVID nurses (Dunn’s test *p* = 0.0021).

More than 50% of the sample reported low levels of depersonalization, except for COVID nurses and physicians, who reported high levels of depersonalization, respectively, in 31.05% and 36.73% of the cases. From the post-hoc analysis, significant differences in the distribution of depersonalization scores (see Figure S10) emerged when comparing COVID physicians to non-COVID physicians (Dunn’s test *p* = 0.0061), other COVID healthcare workers (Dunn’s test *p* = 0.0101), and clerks (Dunn’s test *p* < 0.0001). Moreover, non-COVID physicians differed from other non-COVID healthcare workers (Dunn’s test *p* < 0.0001); COVID nurses reported different levels of depersonalization compared to non-COVID nurses (Dunn’s test *p* = 0.0273) and clerks; and finally non-COVID nurses differed from other non-COVID healthcare workers (Dunn’s test *p* = 0.0024).

More than 58% of the sample reported low levels of reduced personal accomplishment, except for other non-COVID healthcare workers and clerks, who reported high levels of reduced personal accomplishment, respectively, in 32.11% and 56.72% of the cases.

Finally, significant differences in the distribution of personal accomplishment scores (see Figure S11) emerged when comparing clerks to all other categories. Moreover, other non-COVID healthcare workers differed from other COVID healthcare workers (Dunn’s test *p* = 0.0011), non-COVID physicians (Dunn’s test *p* = 0.0050), and non-COVID nurses (Dunn’s test *p* = 0.0022).

### 3.6. Working Conditions and Worry

Considering the whole set of scores, significant differences emerged in the distribution of working conditions scores (see Figure S12) when comparing COVID physicians respectively to non-COVID physicians (Dunn’s test *p* < 0.0001), other COVID healthcare workers (Dunn’s test *p* = 0.0141), and clerks (Dunn’s test *p* = 0.0118); moreover COVID nurses reported different working condition scores compared to non-COVID nurses (Dunn’s test *p* < 0.0001) and other COVID healthcare workers (Dunn’s test *p* = 0.0036); finally differences emerged between non-COVID nurses and other non-COVID healthcare workers (Dunn’s test *p* = 0.0011), and between other non-COVID healthcare workers and clerks (Dunn’s test *p* = 0.0010). Finally, there was a significant difference in the distribution of worry scores (see Figure S13) between non-COVID nurses and non-COVID physicians (Dunn’s test *p* = 0.0250).

## 4. Discussion

The present study investigated short-term psychological consequences of the COVID-19 outbreak in a large sample of Italian healthcare workers. To our knowledge, this is the first study comparing specific subgroups of healthcare workers involved in the management of the pandemic that took into account both their profession and ward (i.e., COVID ward vs. non-COVID ward).

In general, the scores obtained by our sample in depression, anxiety, stress, and insomnia scales can be classified as low according to the cut-off values identified in the literature [26,30]. However, their burnout levels can be classified as medium and high, especially concerning physicians’ and nurses’ scores [36].

Our findings highlighted significant differences among the different groups of healthcare workers. In general, healthcare workers in COVID wards experienced higher levels of anger, anxiety, insomnia, burnout, and PTSD symptoms compared to those of healthcare workers in non-COVID wards. Moreover, nurses were the group at higher risk, as nurses who worked both in COVID and non-COVID wards experienced higher levels of distress than did the other groups of healthcare workers.

Specifically, both physicians and nurses who worked in COVID wards reported worse working conditions, higher depersonalization symptoms, greater states of anger, and a higher tendency to express it compared to those reported by physicians and nurses who worked in non-COVID wards. Moreover, physicians and nurses who worked in COVID wards reported worse working conditions even compared to other healthcare workers who worked in COVID wards. These results are in line with the literature showing that working in COVID wards is an independent risk factor for higher levels of anxiety, burnout, insomnia, and PTSD symptoms [7,18,19,22,25]. Indeed, working in COVID wards entails the risk of contracting the infection and spending several hours under uncomfortable working conditions without taking a break [7]. Moreover, healthcare workers had to treat a large number of patients with limited resources and little knowledge of the disease, which may have resulted in a sense of helplessness among the professionals.

Furthermore, our results showed that non-COVID nurses reported higher levels of worry and PTSD symptoms than did physicians, but in COVID wards these differences were no longer significant. In general, nurses displayed higher insomnia and anxiety symptoms than did physicians in COVID and non-COVID wards. Furthermore, nurses who worked in non-COVID wards had greater anxiety and PTSD symptoms than did other healthcare workers who worked in non-COVID wards. Consistently, the literature underlines working in nursing staff as a risk factor for higher levels of anxiety, insomnia, and PTSD symptoms [5,19,21,37].

The greater distress levels found in nurses can be explained by the longer time spent taking care of patients compared to any other group of healthcare workers. Moreover, they are more frequently exposed to death and patients’ pain. This is even accentuated by the absence of their relatives, who are not allowed in the hospital due to COVID-19 restrictions [19,38,39]. Moreover, the use of protective equipment may be especially relevant to nurses who are frequently in contact with patients and are now prevented from touching distressed and sometimes fatally ill people.

Concerning other healthcare workers, working in COVID wards was associated with higher levels of personal accomplishment compared to working in non-COVID wards, probably because all the healthcare worker who worked in COVID wards felt useful and actively contributing to the health emergency, as also shown in Barello and colleagues’ study [40]. Although other non-COVID healthcare workers seemed to feel less accomplished, they were at lower risk of burnout compared to non-COVID physicians and nurses, probably because they had better working conditions, at least compared to those of nurses.

As far as clerks are concerned, their distress levels did not significantly differ from those of the other groups of healthcare workers who worked in non-COVID wards. Moreover, clerks reported better working conditions, lower distress levels, and lower personal accomplishment compared to the healthcare workers who worked in COVID wards. This is in contrast with a study conducted during the main peak of COVID-19 showing higher levels of anxiety symptoms in nonmedical healthcare workers compared to medical healthcare workers [23]. However, this difference could be due to the fact that their sample of nonmedical healthcare workers included both allied healthcare professionals and clerical staff.

## 5. Conclusions

In conclusion, the baseline findings of the “Healthcare workers’ wellbeing (Benessere Operatori)” project identified nurses in general and healthcare workers operating in COVID wards as vulnerable categories. Despite the sub-threshold distress scores obtained by our sample, the pandemic placed a heavy burden on healthcare workers and the healthcare system in general. Thus, it is essential to keep their distress levels monitored over time, paying particular attention to high-risk individuals. Consistently, policymakers should allocate funds for preventative interventions for these workers’ mental health and reduce mental health stigma in clinical workplaces [41].

The present study illustrated a first profile of psychological responses to the COVID-19 situation in Italian healthcare workers. With the analyses of data derived from the next phases of the project, we will link socio-demographic characteristics, job-related variables, perceived social support, and coping strategies with psychological distress in response to the COVID-19 situation to identify risk and protective factors.

Our findings may assist government advisors and hospitals in providing targeted interventions for healthcare professionals in the face of the COVID-19 outbreak in Italy and other countries.

## Figures and Tables

**Table 1:** The Kruskal–Wallis test results.

Variable	Non-COVID Physicians		COVID Physicians		Non-COVID Nurses		COVID Nurses		Non-COVID Other		COVID Other		Clerks		*p*-Value
	Median	IQR	Median	IQR	Median	IQR	Median	IQR	Median	IQR	Median	IQR	Median	IQR	
DASS_Depression	6	(2,14)	8	(2,14)	8	(2,14)	8	(2,14)	6	(2,12)	6	(2,12)	5	(2,14)	0.1381
DASS_Anxiety	2	(0,6.5)	2	(0,8)	5	(2,10)	6	(2,12)	3	(0,6)	4	(2,12)	4	(2,8)	<0.0001
DASS_Stress	14	(8,20)	14	(10,22)	16	(6,22)	16	(8,22)	12	(8,18)	14	(8,21)	14	(6,20)	0.0275
ISI_Total score	7	(5,10)	8	(5,10)	8.5	(6,11.25)	10	(7,12)	7	(5,10)	9	(5.5,11)	7.5	(6,11)	<0.0001
IES_Intrusion	0.5	(0.12,1.03)	1	(0.38,1.38)	0.88	(0.38,1.75)	1	(0.5,2)	0.5	(0.25,1.12)	0.88	(0.5,1.69)	0.62	(0.25,1.25)	<0.0001
IES_Avoidance	0.5	(0.12,1)	0.75	(0.38,1.12)	0.88	(0.38,1.53)	0.88	(0.38,1.59)	0.62	(0.12,1.12)	0.88	(0.25,1.12)	0.62	(0.38,1.22)	<0.0001
IES_Hyperarousal	0.67	(0.29,1.17)	0.67	(0.33,1.17)	0.83	(0.5,1.83)	0.92	(0.5,2)	0.67	(0.33,1.17)	0.83	(0.25,1.58)	0.67	(0.33,1.33)	0.0003
STAXI_State-Anger	16	(15,19)	18	(16,21)	16.5	(15,22)	18	(15,24)	17	(15,21)	17	(16,22)	17	(15,22)	0.0012
STAXI_Feeling Angry	6	(5,8)	7	(6,9)	6	(5,8)	7	(5,9)	6	(5,8)	7	(5,8)	7	(5,8.75)	0.0558
STAXI_Verbal Exp.	5	(5,6)	5	(5,7)	5	(5,8)	6	(5,9)	5	(5,7)	5	(5,7)	5	(5,7)	<0.0001
STAXI_Physical Exp.	5	(5,5)	5	(5,5)	5	(5,5)	5	(5,6)	5	(5,5)	5	(5,5)	5	(5,5.75)	<0.0001
MBI_Emotional Exhaustion	17	(8,29)	19	(12,29)	14	(8,31.25)	17.5	(9,30)	11	(6,19)	13	(6,25)	15	(7,29.75)	<0.0001
MBI_Depersonalization	4	(1,8.25)	5.5	(3,11.75)	3	(0,8)	6	(2,10)	1	(0,4)	3	(0,7)	2	(0,6.75)	<0.0001
MBI_Personal Accomplishment	39	(31,44)	38.5	(32.25,42.75)	39	(34,44)	39	(35,43.75)	35	(26,42)	41	(36,45)	25	(11.25,36.75)	<0.0001
Working conditions	2.5	(2,3.14)	3.14	(2.6,3.64)	2.67	(2.17,3.3)	3.17	(2.67,3.64)	2.33	(1.86,2.83)	2.5	(2.15,3.17)	2.71	(2.21,3.33)	<0.0001
Worry	4.17	(3.83,4.5)	4.33	(4,4.57)	4.33	(4,4.67)	4.33	(4,4.67)	4.33	(4,4.5)	4.17	(3.83,4.5)	4.17	(4,4.67)	0.0403

**Table 2:** Distress levels distribution according to the cut-off values reported in the literature.

Variable	Non-COVID Physicians	COVID Physicians	Non-COVID Nurses	COVID Nurses	Non-COVID Other	COVID Other	Clerks
DASS_Depression							
normal (0–9)	130 (65%)	56 (57.14%)	100 (58.14%)	102 (53.68%)	142 (65.14%)	26 (60.47%)	80 (59.7%)
mild (10–12)	19 (9.5%)	13 (13.27%)	18 (10.47%)	32 (16.84%)	28 (12.84%)	10 (23.26%)	19 (14.18%)
moderate (13–20)	33 (16.5%)	15 (15.31%)	33 (19.19%)	29 (15.26%)	31 (14.22%)	3 (6.98%)	22 (16.42%)
severe (21–27)	7 (3.5%)	7 (7.14%)	14 (8.14%)	12 (6.32%)	12 (5.5%)	1 (2.33%)	7 (5.22%)
extremely severe depression (28–42)	11 (5.5%)	7 (7.14%)	7 (4.07%)	15 (7.89%)	5 (2.29%)	3 (6.98%)	6 (4.48%)
DASS_Anxiety							
normal (0–6)	150 (75%)	71 (72.45%)	102 (59.3%)	107 (56.32%)	168 (77.06%)	27 (62.79%)	92 (68.66%)
mild (7–9)	15 (7.5%)	8 (8.16%)	9 (5.23%)	19 (10%)	15 (6.88%)	2 (4.65%)	11 (8.21%)
moderate (10–14)	22 (11%)	13 (13.27%)	27 (15.7%)	25 (13.16%)	16 (7.34%)	5 (11.63%)	18 (13.43%)
severe (15–19)	10 (5%)	2 (2.04%)	13 (7.56%)	15 (7.89%)	10 (4.59%)	2 (4.65%)	7 (5.22%)
extremely severe anxiety (20–42)	3 (1.5%)	4 (4.08%)	21 (12.21%)	24 (12.63%)	9 (4.13%)	7 (16.28%)	6 (4.48%)
DASS_Stress							
normal (0–10)	72 (36%)	31 (31.63%)	66 (38.37%)	65 (34.21%)	103 (47.25%)	19 (44.19%)	56 (41.79%)
mild (11–18)	77 (38.5%)	34 (34.69%)	51 (29.65%)	60 (31.58%)	61 (27.98%)	10 (23.26%)	41 (30.6%)
moderate (19–26)	35 (17.5%)	21 (21.43%)	32 (18.6%)	32 (16.84%)	38 (17.43%)	10 (23.26%)	24 (17.91%)
severe (27–34)	13 (6.5%)	7 (7.14%)	16 (9.3%)	22 (11.58%)	15 (6.88%)	2 (4.65%)	9 (6.72%)
extremely severe stress (35–42)	3 (1.5%)	5 (5.1%)	7 (4.07%)	11 (5.79%)	1 (0.46%)	2 (4.65%)	4 (2.99%)
ISI_Total score							
absence of insomnia (0–7)	119 (59.5%)	48 (48.98%)	76 (44.19%)	58 (30.53%)	118 (54.13%)	17 (39.53%)	67 (50%)
sub-threshold insomnia (8–14)	78 (39%)	45 (45.92%)	75 (43.6%)	106 (55.79%)	84 (38.53%)	22 (51.16%)	58 (43.28%)
moderate insomnia (15–21)	3 (1.5%)	5 (5.1%)	21 (12.21%)	25 (13.16%)	16 (7.34%)	3 (6.98%)	9 (6.72%)
severe insomnia (22–28)	0 (0%)	0 (0%)	0 (0%)	1 (0.53%)	0 (0%)	1 (2.33%)	0 (0%)
MBI_Emotional Exhaustion							
low (≤14)	85 (42.5%)	36 (36.73%)	88 (51.16%)	71 (37.37%)	143 (65.6%)	22 (51.16%)	65 (48.51%)
medium (15–23)	46 (23%)	25 (25.51%)	21 (12.21%)	52 (27.37%)	33 (15.14%)	9 (20.93%)	28 (20.9%)
high (≥24)	69 (34.5%)	37 (37.76%)	63 (36.63%)	67 (35.26%)	42 (19.27%)	12 (27.91%)	41 (30.6%)
MBI_Depersonalization							
low (≤4)	99 (49.5%)	29 (29.59%)	88 (51.16%)	69 (36.32%)	151 (69.27%)	23 (53.49%)	79 (58.96%)
medium (4–8)	51 (25.5%)	33 (33.67%)	44 (25.58%)	62 (32.63%)	47 (21.56%)	13 (30.23%)	27 (20.15%)
high (≥9)	50 (25%)	36 (36.73%)	40 (23.26%)	59 (31.05%)	20 (9.17%)	7 (16.28%)	28 (20.9%)
MBI_Personal Accomplishment							
low (≥37)	117 (58.5%)	58 (59.18%)	104 (60.47%)	122 (64.21%)	101 (46.33%)	30 (69.77%)	34 (25.37%)
medium (30–36)	45 (22.5%)	24 (24.49%)	37 (21.51%)	42 (22.11%)	47 (21.56%)	10 (23.26%)	24 (17.91%)
high (≤29)	38 (19%)	16 (16.33%)	31 (18.02%)	26 (13.68%)	70 (32.11%)	3 (6.98%)	76 (56.72%)

## Data Availability

Data cannot be shared because participants did not provide written informed consent for it.

## Supplementary Materials

The following are available online at https://www.mdpi.com/article/10.3390/ijerph18105267/s1, Figure S1: Dunn’s test for DASS-21 Anxiety, Figure S2: Dunn’s test for ISI TOT, Figure S3: Dunn’s test for IES-R Intrusion, Figure S4: Dunn’s test for IES-R Avoidance, Figure S5: Dunn’s test for IES-R Hyperarousal, Figure S6: Dunn’s test for State-Anger, Figure S7: Dunn’s test for STAXI Verbal Expression, Figure S8: Dunn’s test for STAXI Physical Expression, Figure S9: Dunn’s test for MBI Emotional Exhaustion, Figure S10: Dunn’s test for MBI Depersonalization, Figure S11: Dunn’s test for MBI Personal Accomplishment, Figure S12: Dunn’s test for Working Condition, Figure S13: Dunn’s test for Worry.
