# Supplementary figures and images for: The “Healthcare Workers’ Wellbeing (Benessere Operatori)” Project: A Picture of the Mental Health Conditions of Italian Healthcare Workers during the First Wave of the COVID-19 Pandemic
Source: Int J Environ Res Public Health. 2021 May 15;18(10):5267. doi: 10.3390/ijerph18105267 (PMC8156728; doi:10.3390/ijerph18105267)

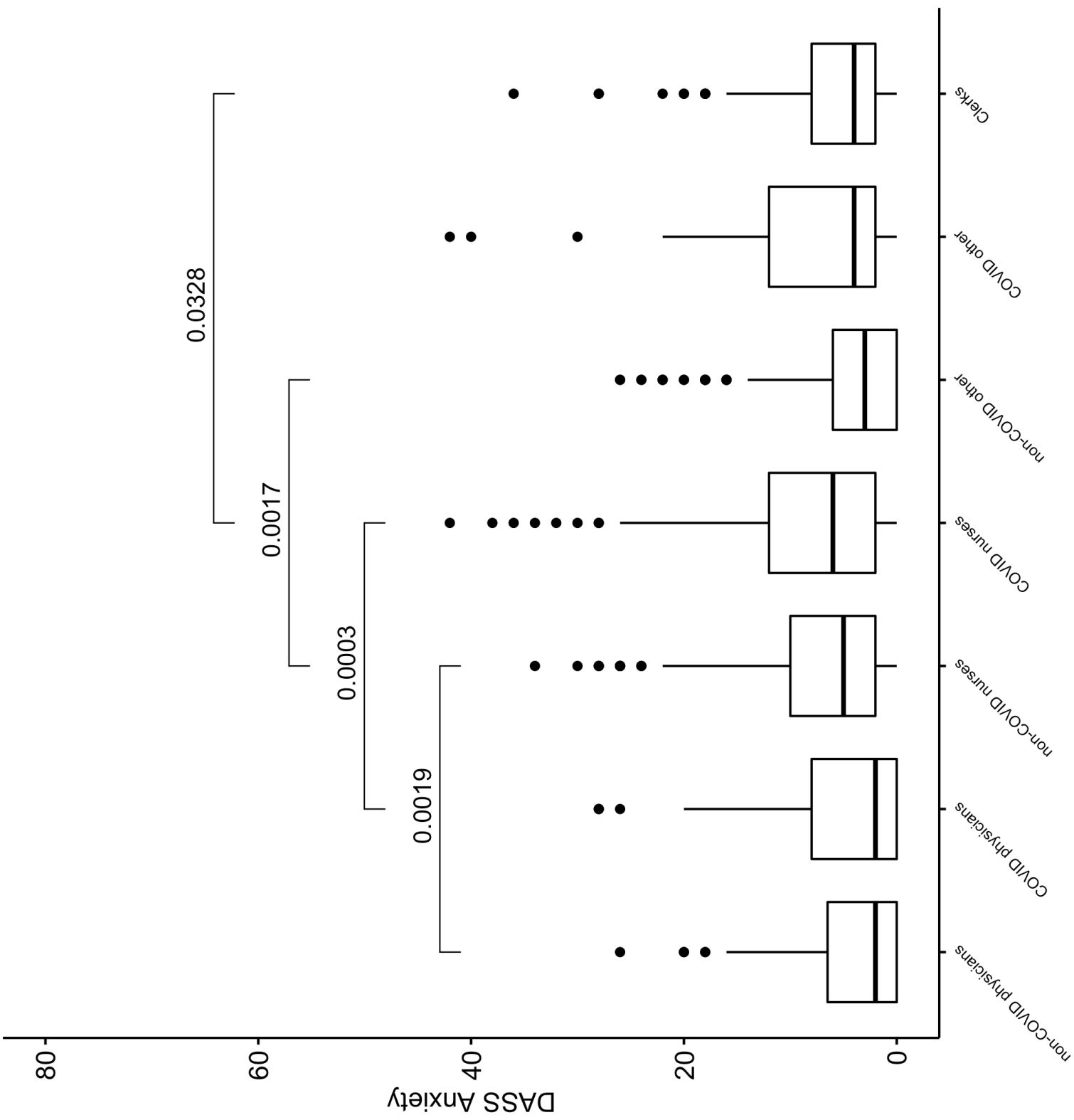

Supplement: Supplementary file 1 [file ijerph-18-05267-s001.zip › S1 Dunn's test for DASS-21 Anxiety.pdf]

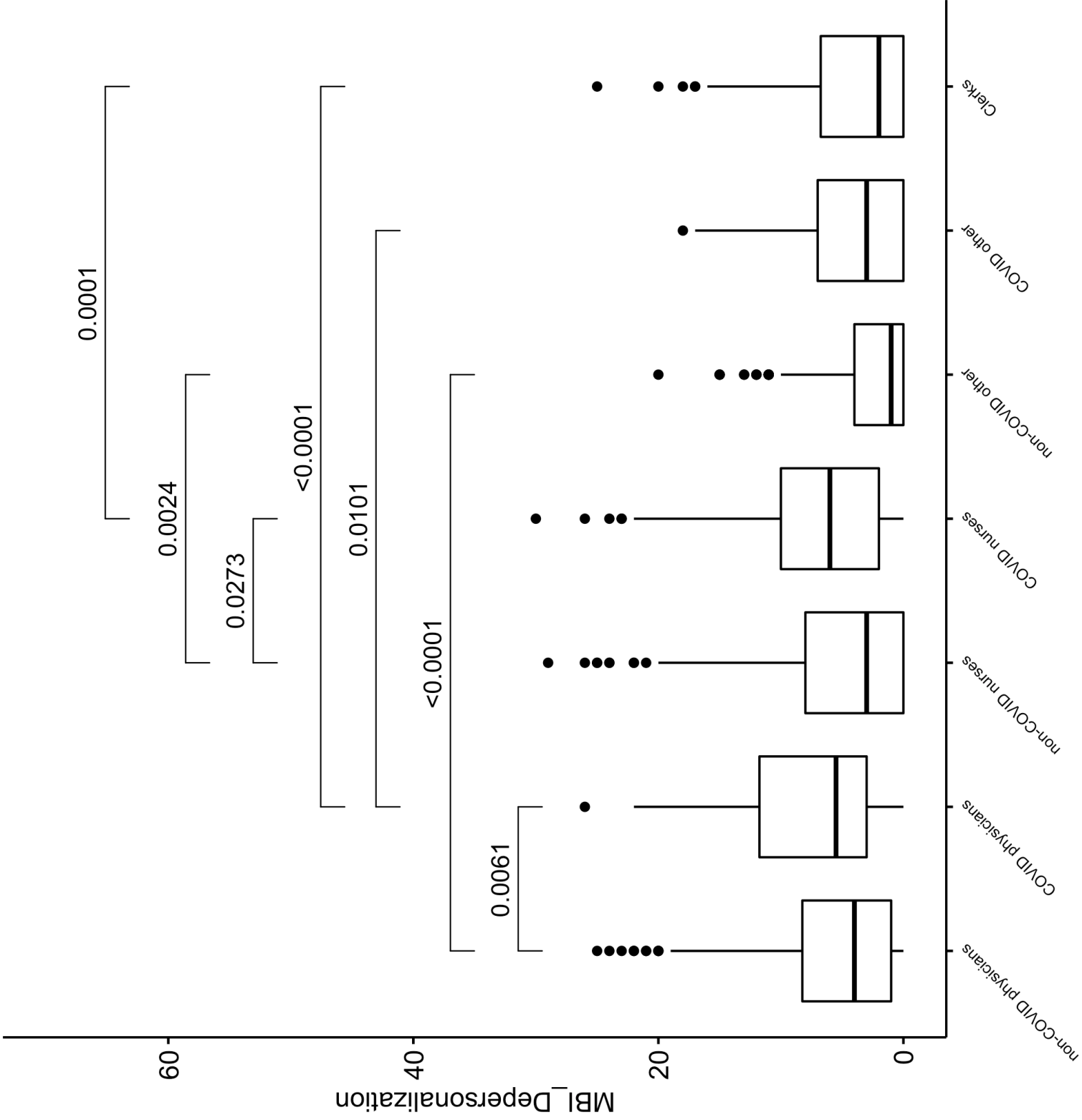

Supplement: Supplementary file 1 [file ijerph-18-05267-s001.zip › S10 Dunn's test for MBI Depersonalization.pdf]

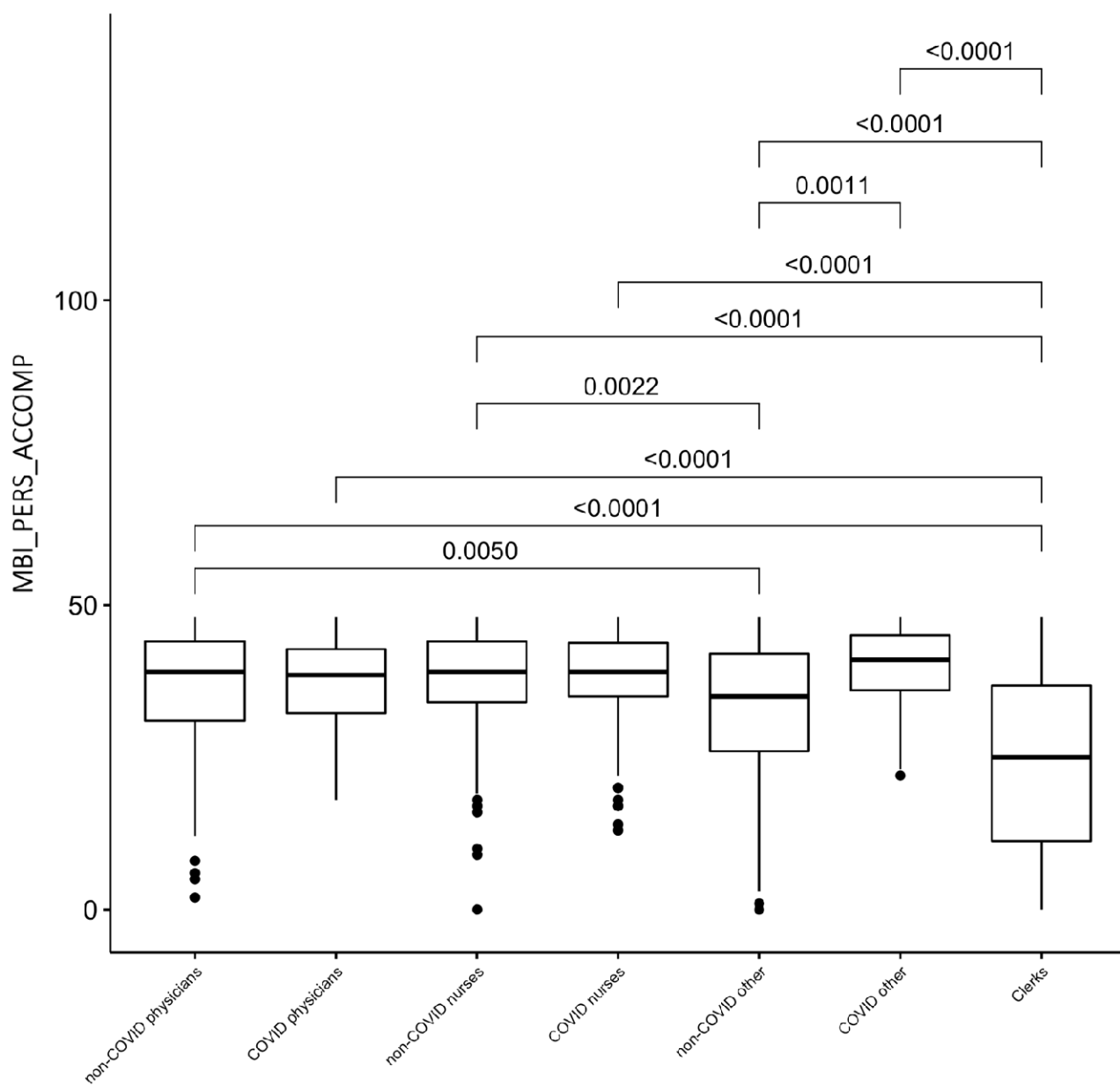

Supplement: Supplementary file 1 [file ijerph-18-05267-s001.zip › S11 Dunn's test for MBI Personal Accomplishment.pdf]

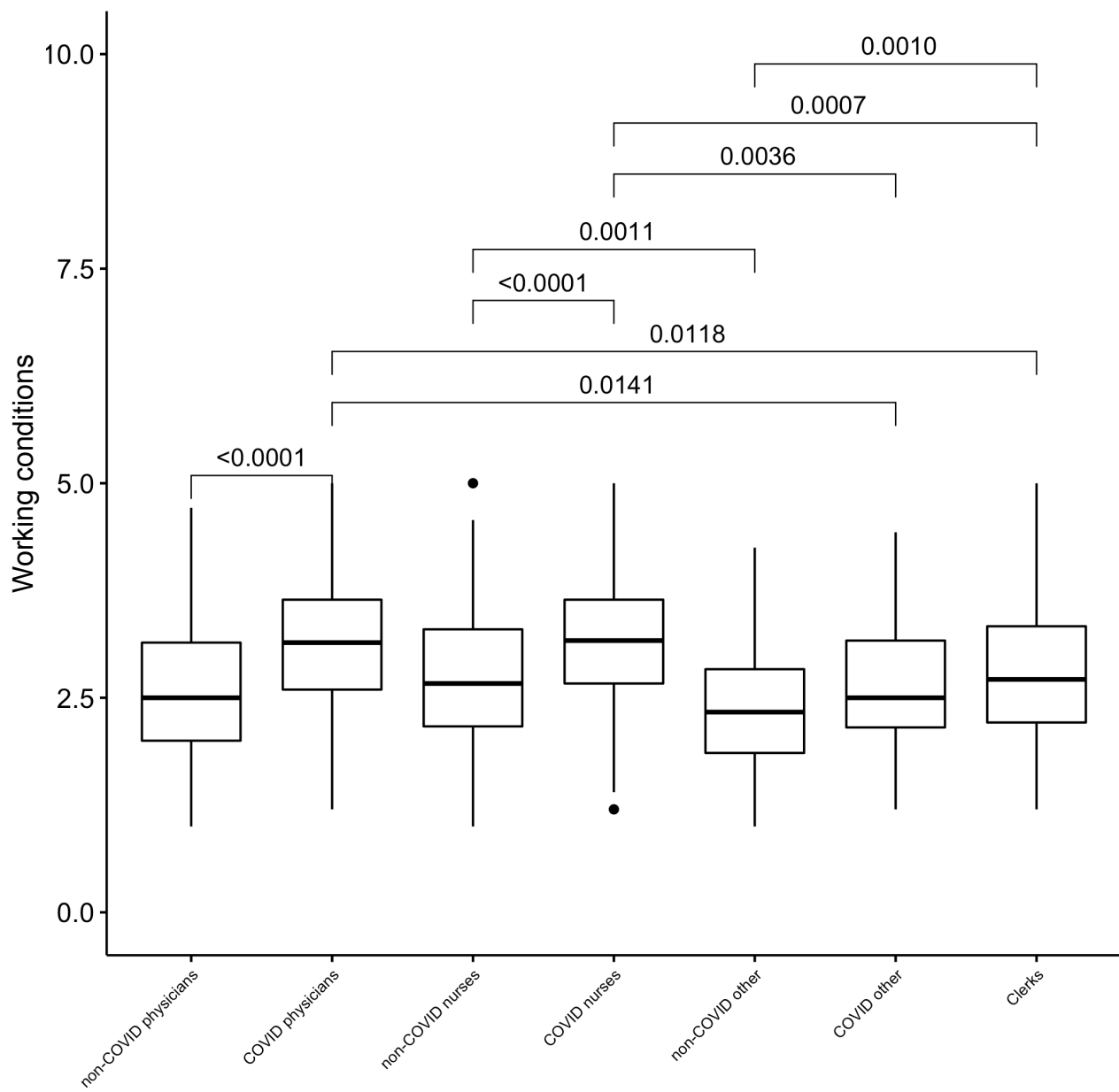

Supplement: Supplementary file 1 [file ijerph-18-05267-s001.zip › S12 Dunn's test for Working Conditions.pdf]

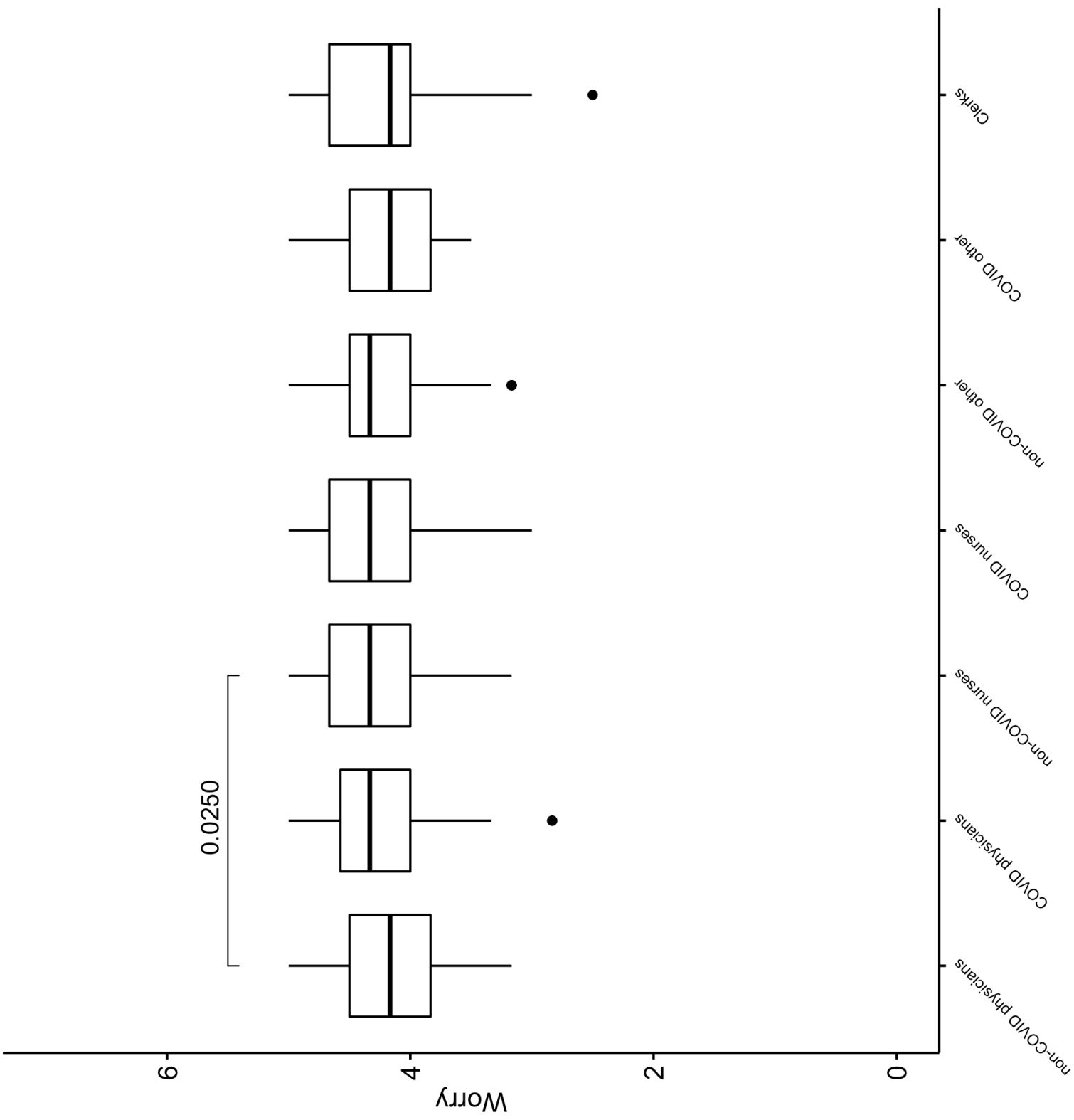

Supplement: Supplementary file 1 [file ijerph-18-05267-s001.zip › S13 Dunn's test for Worry.pdf]

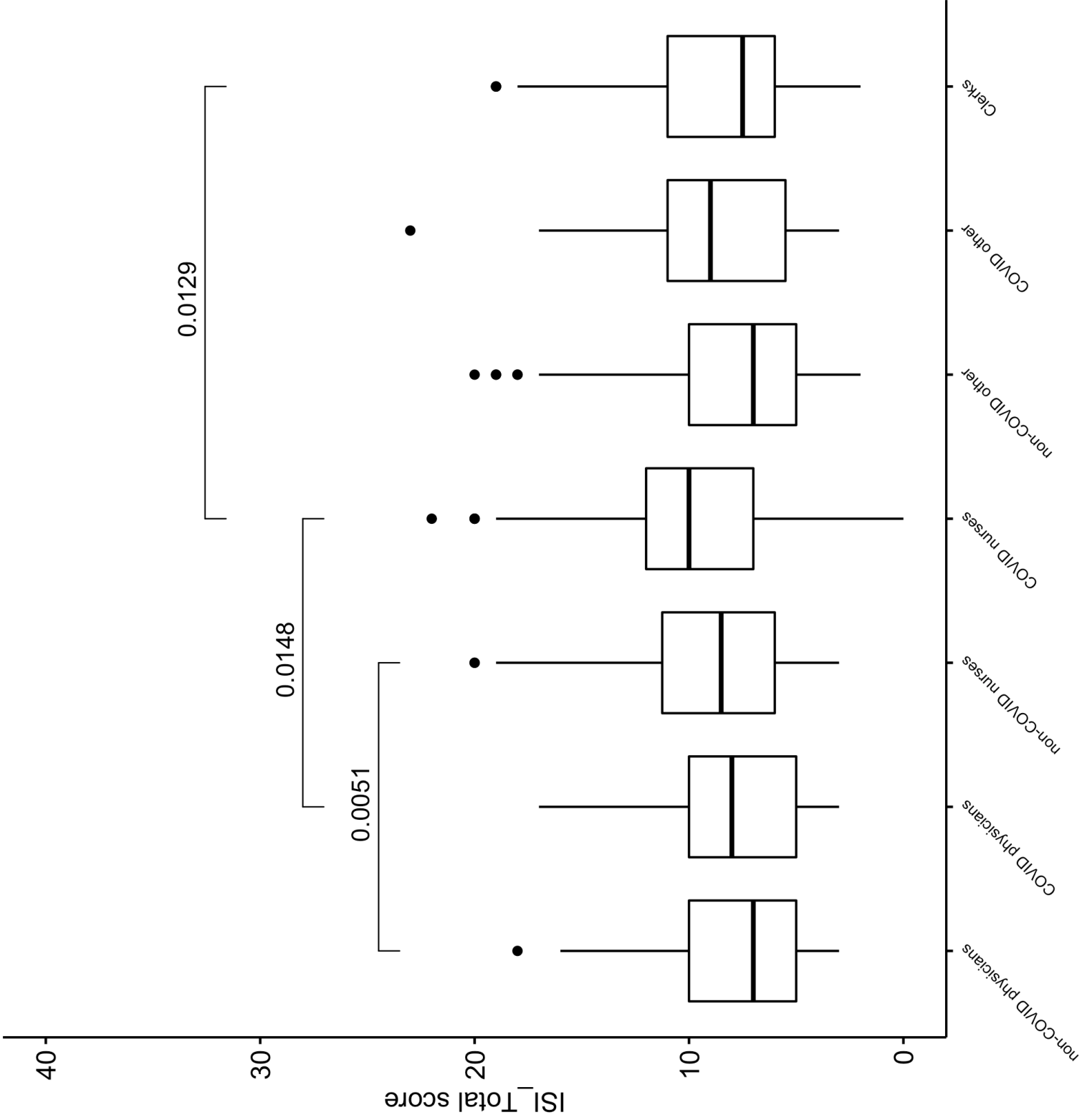

Supplement: Supplementary file 1 [file ijerph-18-05267-s001.zip › S2 Dunn's test for ISI TOT.pdf]

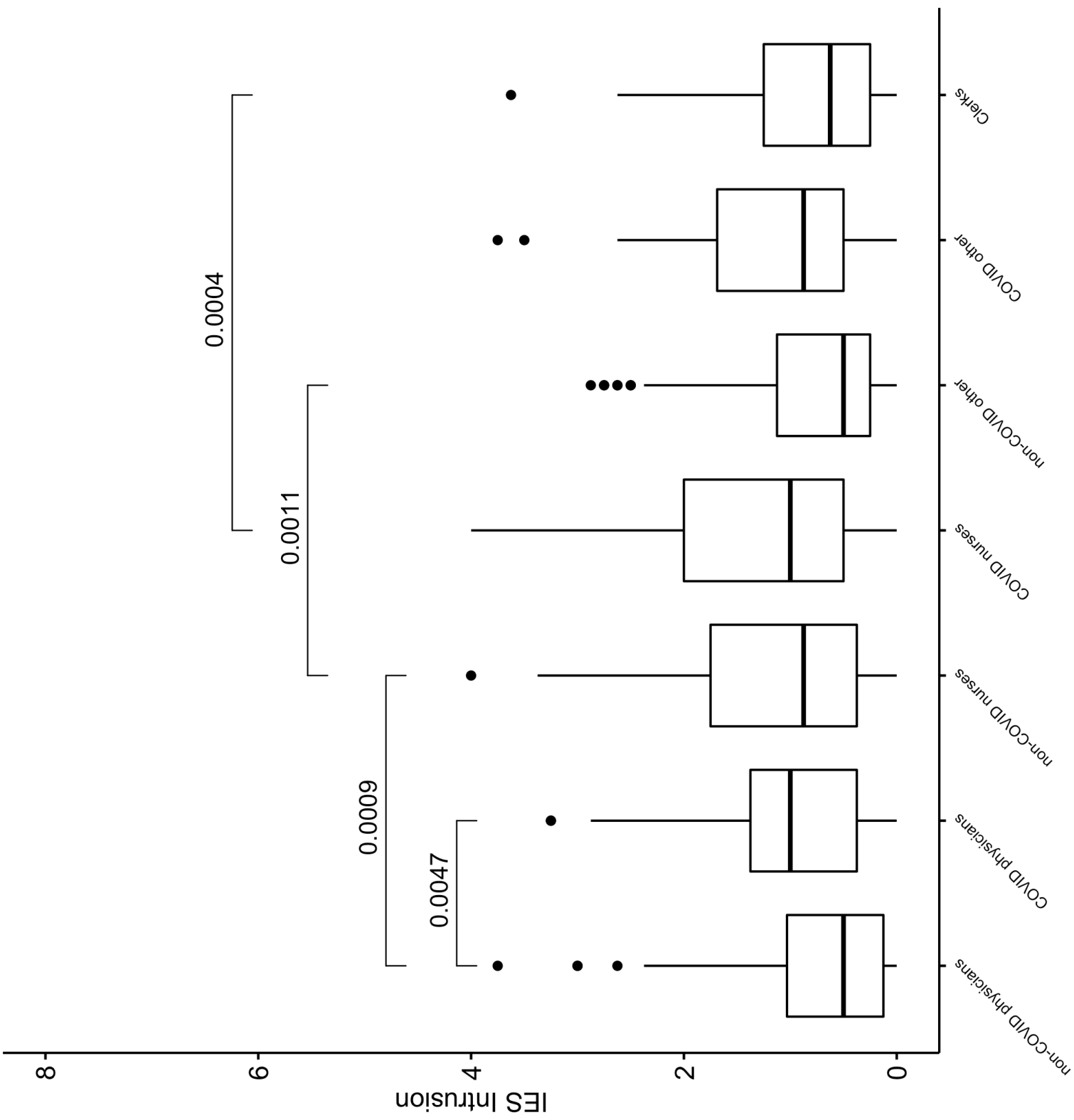

Supplement: Supplementary file 1 [file ijerph-18-05267-s001.zip › S3 Dunn's test for IES-R Intrusion.pdf]

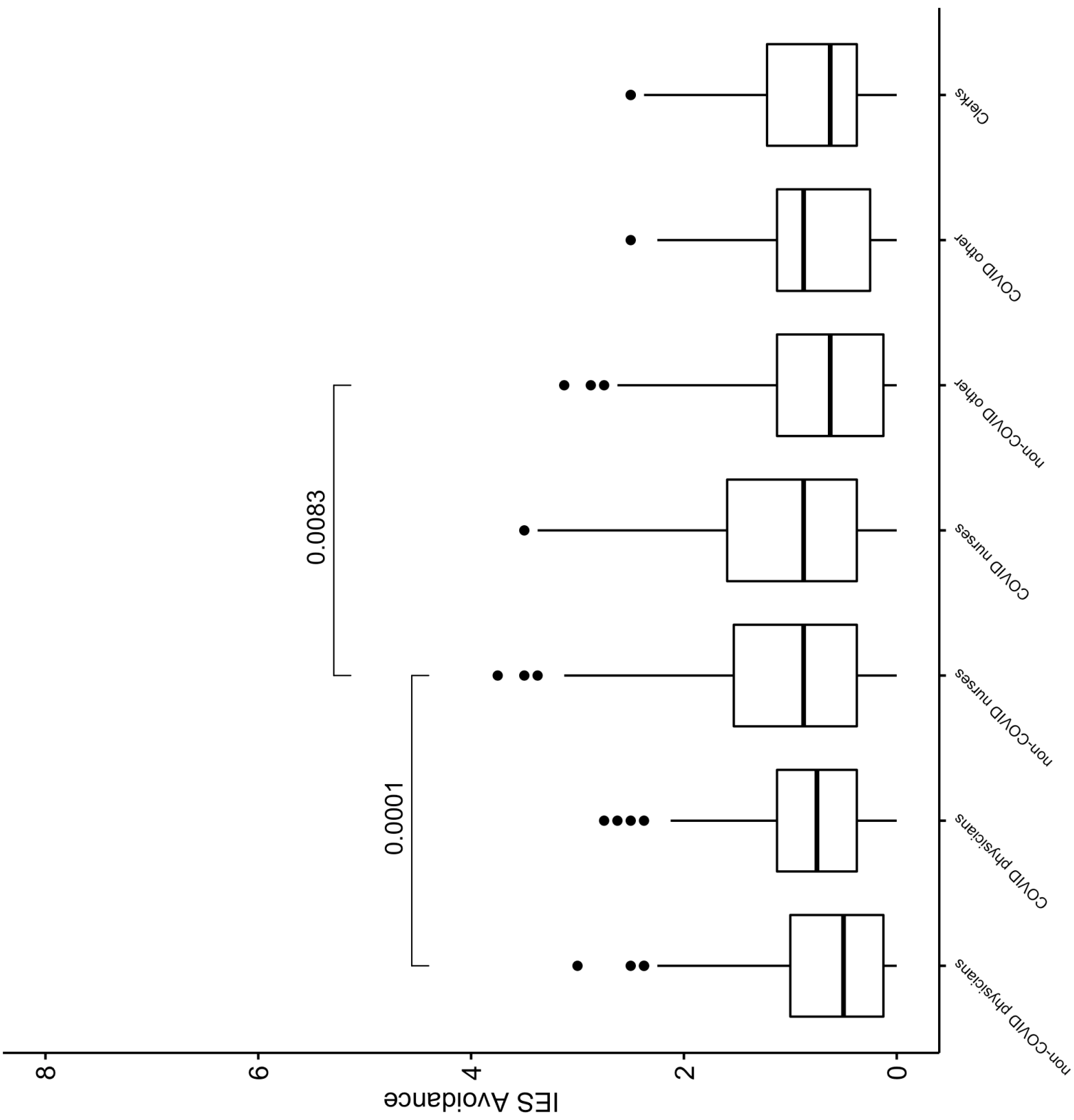

Supplement: Supplementary file 1 [file ijerph-18-05267-s001.zip › S4 Dunn's test for IES-R Avoidance.pdf]

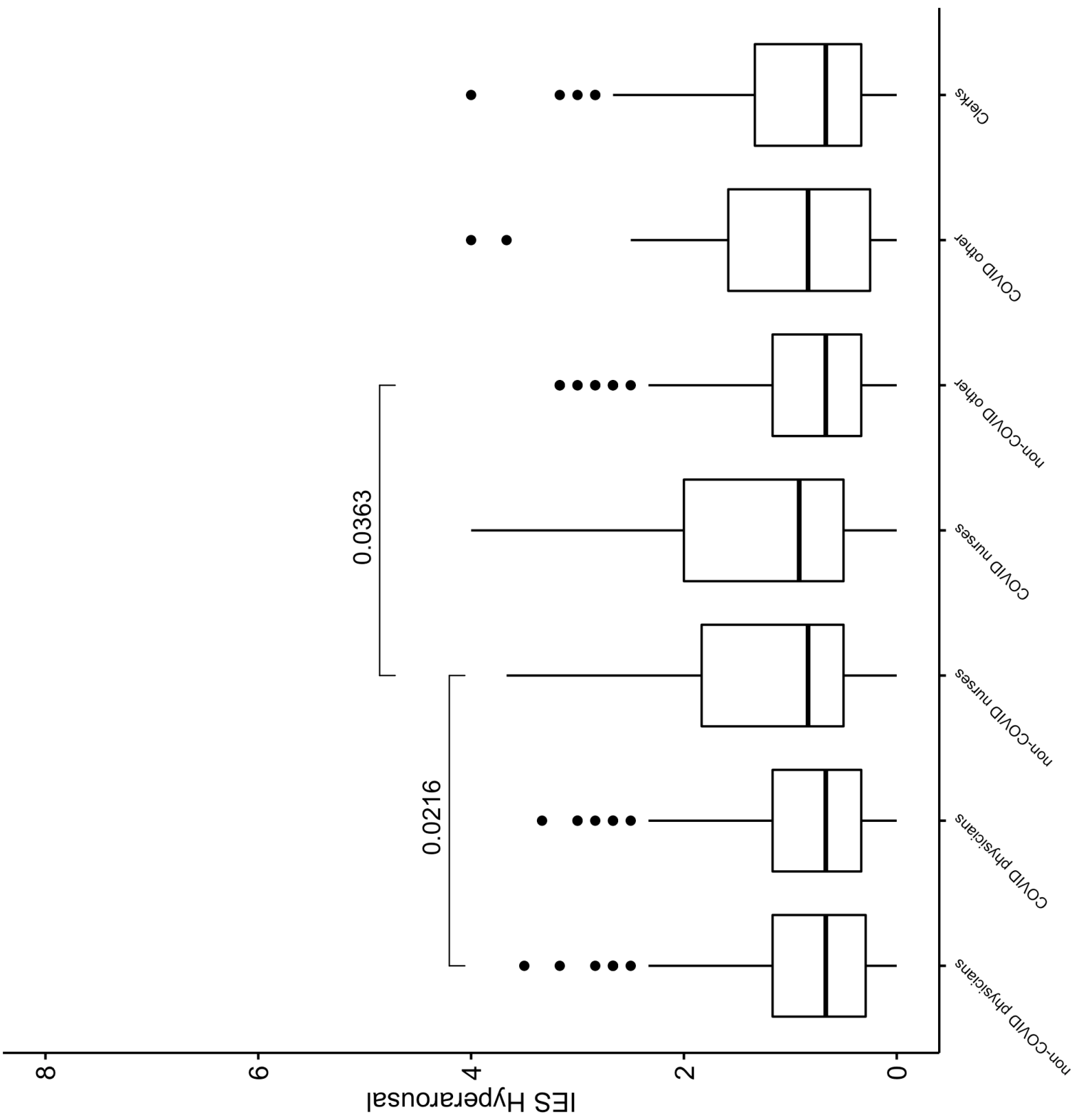

Supplement: Supplementary file 1 [file ijerph-18-05267-s001.zip › S5 Dunn's test for IES-R Hyperarousal.pdf]

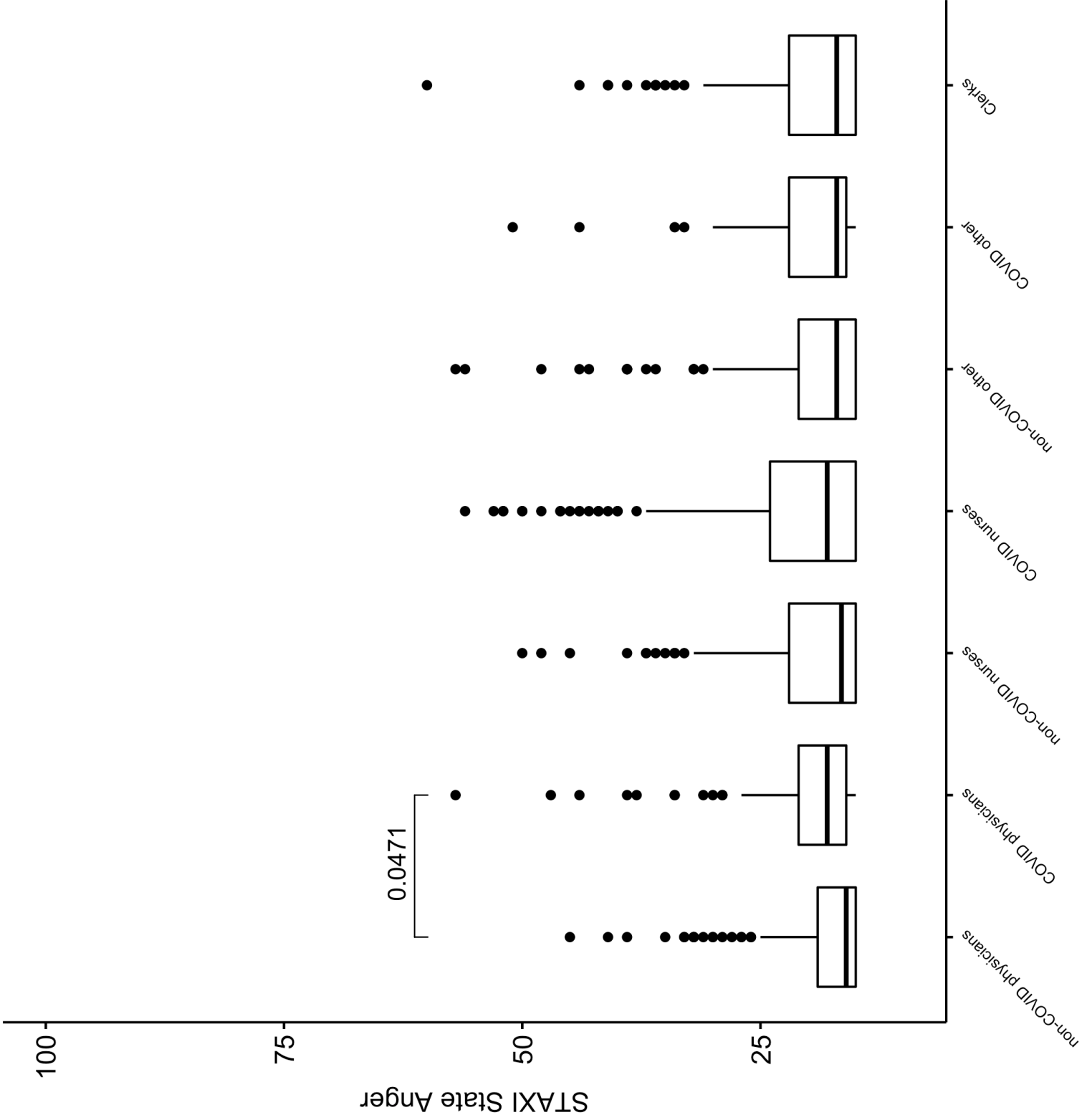

Supplement: Supplementary file 1 [file ijerph-18-05267-s001.zip › S6 Dunn's test for State Anger.pdf]

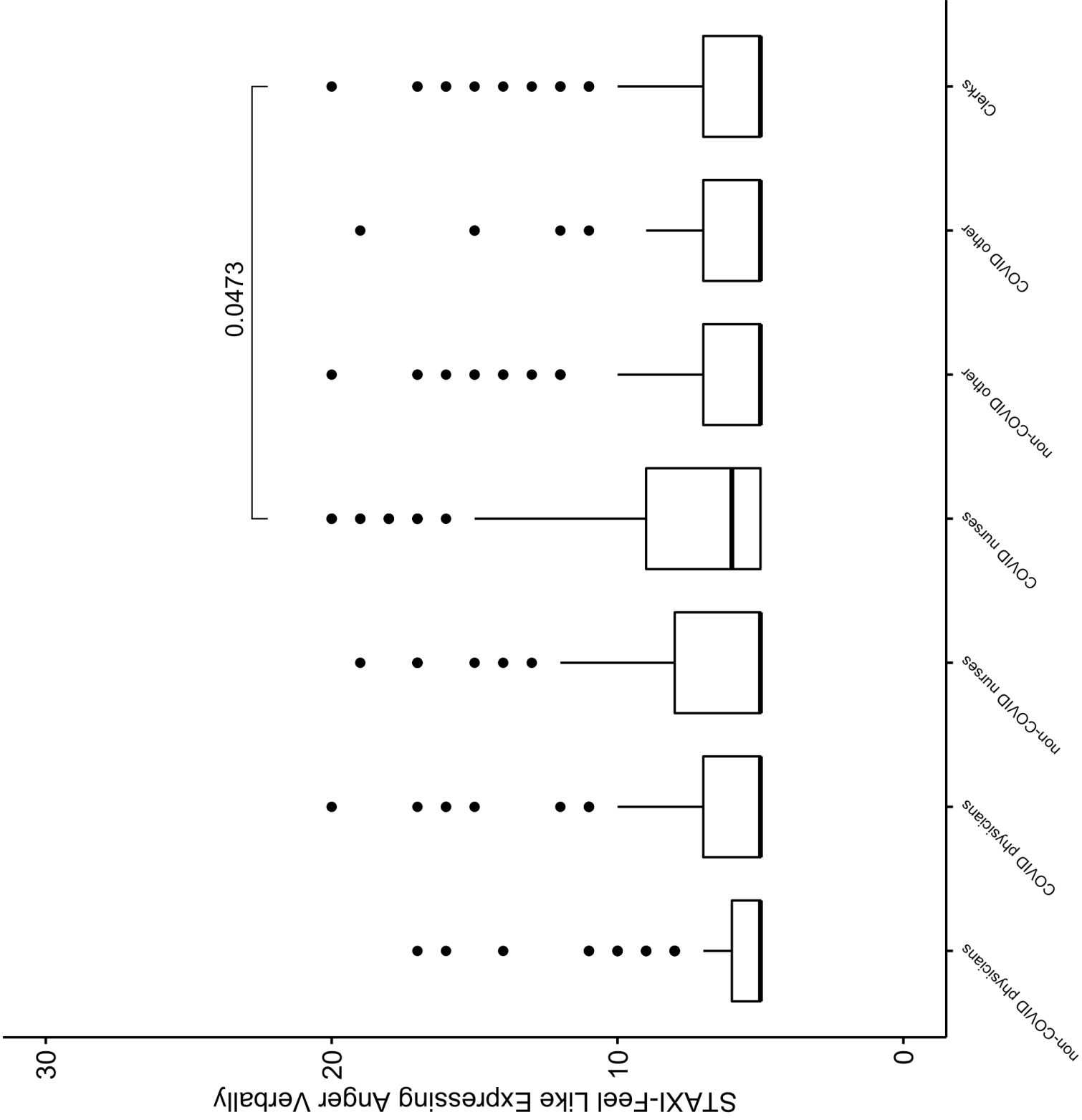

Supplement: Supplementary file 1 [file ijerph-18-05267-s001.zip › S7 Dunn's test for STAXI Verbal Expression.pdf]

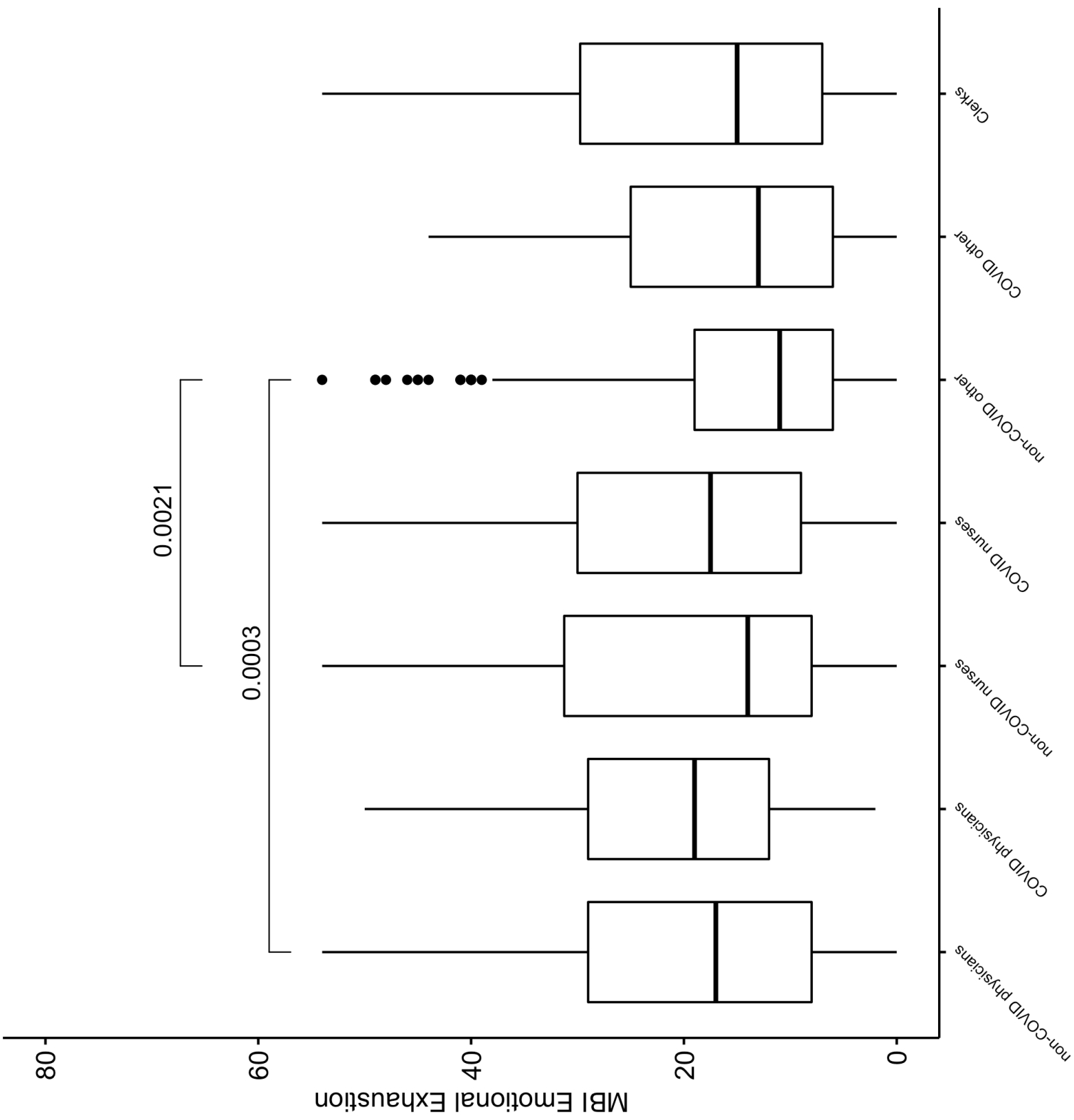

Supplement: Supplementary file 1 [file ijerph-18-05267-s001.zip › S9 Dunn's test for MBI Emotional Exhaustion.pdf]
