# Supplementary material for: The “Healthcare Workers’ Wellbeing (Benessere Operatori)” Project: A Picture of the Mental Health Conditions of Italian Healthcare Workers during the First Wave of the COVID-19 Pandemic
Source: Int J Environ Res Public Health. 2021 May 15;18(10):5267. doi: 10.3390/ijerph18105267 (PMC8156728; doi:10.3390/ijerph18105267)
Supplement: Supplementary file 1 [file S8 Dunn's test for STAXI Physical Expression.pdf › S8 Dunn's test for STAXI Physical Expression.pdf]

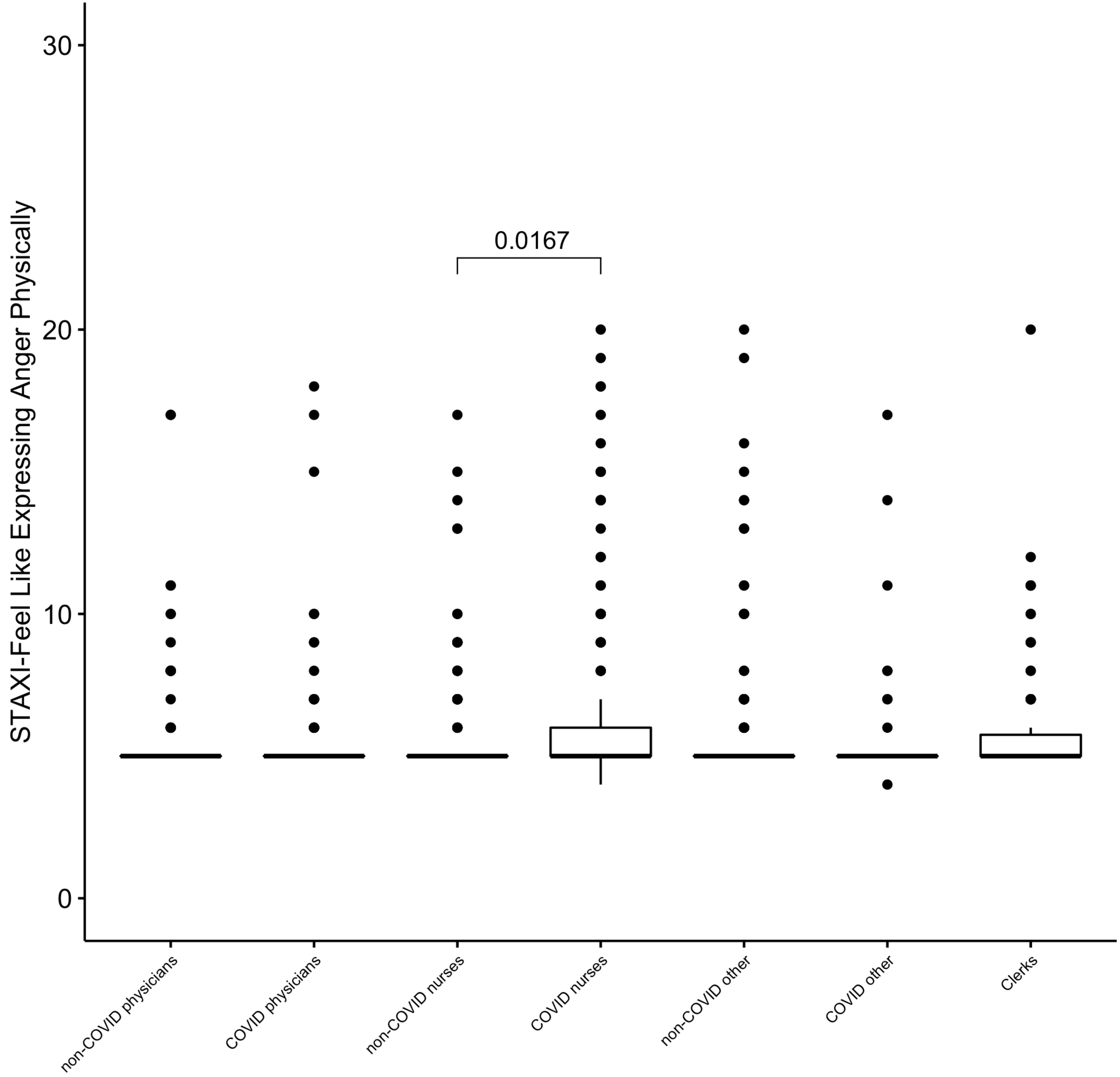
0.0167
STAXI-Feel Like Expressing Anger Physically
30
20
10
0
non-COVID physicians
COVID physicians
non-COVID nurses
COVID nurses
non-COVID other
COVID other
Clerks
